# Characteristics of Gastric Endoscopic and Histopathological Findings in the South and Southeast Asian Populations

**DOI:** 10.1002/jgh3.70242

**Published:** 2025-09-22

**Authors:** Mitsushige Sugimoto, Takeshi Matsuhisa, Hafeza Aftab, Sirikan Limpakan, Sunil K. Sharma Dhakal, Kim Sang, Kyaw Htet, Than Than Yee, Yoshio Yamaoka

**Affiliations:** ^1^ Division of Genome‐Wide Infectious Microbiology, Research Center for GLOBAL and LOCAL Infectious Disease Oita University Yufu Japan; ^2^ Department of Gastroenterology St. Marianna University School of Medicine Kawasaki Japan; ^3^ Department of Gastroenterology Dhaka Medical College Dhaka Bangladesh; ^4^ Gastrointestinal Surgery and Endoscopy Faculty of Medicine, Chiang Mai University Chiang Mai Thailand; ^5^ Digestive Disease Center, Nepal Mediciti Nakhu Kathmandu Nepal; ^6^ Department of Endoscopy and Gastroenterology City International Hospital Ho Chi Minh City Vietnam; ^7^ Department of Surgery Defense Services General Hospital (1/1000) Yangon Myanmar; ^8^ Department of Gastrointestinal and Hepatobiliary Surgery, Defense Services General Hospital (2/1000) Nay Pyi Taw Myanmar; ^9^ Division of Genome‐Wide Infectious Microbiology, Research Center for GLOBAL and LOCAL Infectious Disease, Department of Environmental and Preventive Medicine Oita University Yufu Japan; ^10^ Department of Medicine, Gastroenterology and Hepatology Section Baylor College of Medicine Houston Texas USA

**Keywords:** gastritis, *Helicobacter pylori*, pepsinogen, virulence factor

## Abstract

**Background and Aim:**

*Helicobacter pylori* infection rates and severity and susceptibility to gastric diseases vary widely in different populations because of different *H. pylori* strains, lifestyles, and genetic factors. South and Southeast Asia is a region where many ethnic groups are intermingled, and *H. pylori* strains have been shifting from Western‐type to East Asian‐type strains. We aimed to investigate the different endoscopic and histopathologic features in five South and Southeast Asian countries.

**Methods:**

We examined differences in endoscopic and histopathological gastritis and *H. pylori* infection status in 2426 patients from South and Southeast Asian populations in Vietnam, Thailand, Myanmar, Bangladesh, and Nepal.

**Results:**

Among Asian patients with abdominal symptoms, such as epigastric pain, nausea, abdominal discomfort, constipation, and heartburn, the prevalence of gastric disease, *H. pylori* infection status (current, past, and non‐infection), and severity of histopathological gastritis significantly differed between countries. Less than 10% of patients had atrophy and intestinal metaplasia, irrespective of country and *H. pylori* infection status; their severity when present was also mild. Although patients with gastric cancer had higher rates of atrophy and intestinal metaplasia compared with those with other diseases, half of them had no pathological atrophy or intestinal metaplasia.

**Conclusions:**

Gastric condition and susceptibility to gastric disease differed among South and Southeast Asian populations. *H. pylori* infection rates remain high in Asian countries, but the frequency and severity of atrophy and gastric mucosal atrophy were low.

## 1 Introduction

*Helicobacter pylori* causes chronic gastritis and preneoplastic lesions such as atrophy and intestinal metaplasia in most infected individuals [1, 2, 3, 4]. Gastric cancer develops through a multistep process known as the Correa cascade, in which *H. pylori* infection plays a central role in initiating chronic inflammation that may progress to atrophy, intestinal metaplasia, dysplasia, and ultimately carcinoma. While atrophy and intestinal metaplasia are associated with an increased risk of gastric cancer, they are not the only contributing factors. Other risk factors include high‐salt and smoked diets, low intake of fruits and vegetables, smoking, genetic polymorphisms, and family history of gastric cancer or hereditary cancer syndromes. Surveillance endoscopy decreases the mortality and incidence rates of gastric cancer by decreasing the risk of diagnosis at an advanced stage [5, 6]. Although the gastric condition and susceptibility to gastric cancer in patients with *H. pylori* infection differ widely among populations, the efficacy of screening and surveillance endoscopy for gastric cancer in the South and Southeast Asian populations and the correlation between endoscopic and histopathological evaluations have not been fully discussed.

The genetic diversity of *H. pylori* decreases according to distance from East Africa, where it is thought to have spread along with human migration around 58 000 years ago [7]. Migrations from Asian countries into the Pacific had two routes: migrations to New Guinea and Australia accompanied by hpSahul and those with hspMaori from Taiwan through the Pacific [8]. In addition, human and *H. pylori* strain migration in Southeast Asia included migration from India introducing hpEurope bacteria into Thailand, Cambodia, and Malaysia; migration carrying hspEAsia bacteria into Vietnam and Cambodia; migration from Southern China into Thailand carrying hpAsia2; migration from China to Thailand and Malaysia resulting in the spread of hspEasia strains; and migration from India to Malaysia distributing both hpAsia2 and hpEurope [9]. Along with ethnic migrations, the virulence of *H. pylori* to the gastric mucosa is expected to change gradually; however, even though *H. pylori* strains are similar in the same Asian populations, the incidence of gastric cancer varies widely [10]. It is important to clarify the *H. pylori* situation, the specificity of and susceptibility to gastric disease, and the severity of gastritis in each population.

The “Asian paradox” might be explained by the widespread prevalence of weakly cytotoxic strains and the correspondingly low frequency of *H. pylori* ‐associated diseases [11, 12]. Studies have examined the characteristics of gastritis severity and susceptibility to gastric disease in each Asian country, but no differences in surrounding South and Southeast Asian populations have been assessed using the same endoscopic and pathological criteria. We investigated endoscopic and histopathologic gastric features in five Asian countries. We also aimed to determine optimal cut‐off values for pepsinogen markers in identifying individuals at high risk for *H. pylori* infection, gastric atrophy, and cancer, using ROC analysis and the Youden index.

## 2 Methods

To evaluate the gastric endoscopic and histopathological characteristics of the populations in Vietnam, Thailand, Myanmar, Bangladesh, and Nepal, we enrolled patients with abdominal symptoms, such as epigastric pain, nausea, abdominal discomfort, constipation, and heartburn, from January 1996 to January 2012. Inclusion criteria in this study were patients aged ≥ 20 years with any of the abdominal symptoms. Exclusion criteria were patients who received treatment for *H. pylori* or who had surgery involving the stomach. All patients underwent endoscopy for screening and received a diagnosis of *H. pylori* along with an endoscopic and histopathological diagnosis of gastritis severity. Additionally, serum gastrin and pepsinogen levels on an empty stomach before breakfast were evaluated.

The study protocol conformed to the ethical guidelines of the Declaration of Helsinki, and the ethics committees approved the conduct of this study. Ethical approval was given by the Ethics Committee of the Ethics Committee at Oita University Faculty of Medicine (P‐12‐10, and #1660). Some data used in this study were used in a previous study comparing gastritis characteristics with each country and Japan [13, 14, 15, 16, 17].

### 2.1 Endoscopic Evaluation

Endoscopic examinations were performed using white light imaging (WLI) alone, with endoscopes and equipment that were routinely used in clinical practice at each participating facility. Therefore, the types of endoscopes and equipment varied depending on the time of implementation, country, and hospital.

During endoscopy, the severity of gastritis was assessed using the Updated Sydney System and the Kimura–Takemoto classification [18, 19]. Peptic ulcers and gastric polyps were diagnosed based on endoscopic findings, while gastric cancer was diagnosed based on both endoscopic and histological evaluation, regardless of the degree of gastric mucosal inflammation or atrophy. If gastric cancer was suspected in patients with peptic ulcers, histopathological examination was also performed.

Endoscopic atrophic gastritis was diagnosed when atrophy was classified as C‐II or greater according to the Kimura–Takemoto classification [18]. Endoscopic gastritis was defined as inflammation characterized by redness or edematous mucosa in the absence of atrophy and intestinal metaplasia. Patients without endoscopic evidence of either atrophy or inflammation were classified as normal.

To ensure consistency, one expert endoscopist (MT) reviewed white light images and evaluated the severity of gastritis and the presence of gastric diseases for all patients after the examinations.

### 2.2 Pathological Evaluation of Gastritis

Pathological severity of gastritis, including chronic inflammation, neutrophil activity, atrophy, intestinal metaplasia, and *H. pylori* infection, was scored using a four‐point scale ranging from 0 to 3 (0: none, 1: mild, 2: moderate, and 3: severe) based on the Houston‐updated version of the Sydney system [19]. Biopsy specimens were taken from three sites: the greater curvature of the antrum, the greater curvature of the upper corpus, and the lesser curvature of the lower corpus. One experienced pathologist assessed all histological sections to avoid diagnostic bias and eliminate interobserver variability. The severity of chronic inflammation, neutrophil activity, atrophy, and intestinal metaplasia was evaluated using the Houston‐updated version of the Sydney System, based on biopsies obtained from three sites. For each histological feature, the highest score among the three sites was recorded as the representative value. Furthermore, if a score of 1 or higher was observed in any of the three biopsy sites, the patient was classified as positive for that histological finding.

### 2.3 *H. pylori* Infection Status

Patients with no medical history of *H. pylori* eradication therapy were included in the questionnaire. Infection status was assessed based on the results of a culture test or histological evaluation by hematoxylin and eosin (HE) staining. If *H. pylori* bacteria themselves were observed in the pathological evaluations and there were findings of inflammatory cell infiltration into the gastric mucosa, the pathological examination was considered to be positive for *H. pylori* infection. We divided patients into three groups based on the status of *H. pylori* infection: non‐infection, past infection, and current infection. Patients were considered positive for *H. pylori* infection if at least one of the two detection methods (a culture test or histological evaluation) was positive. Those with negative results for both detection methods along with no pathological atrophy, intestinal metaplasia, and inflammatory cell infiltrations were diagnosed as non‐infection. Those with negative results for both detection methods along with pathological atrophy and intestinal metaplasia and no eradication history were diagnosed as past infection.

### 2.4 Serum Pepsinogen Levels

As a marker of gastric atrophy, serum levels of pepsinogen I and II were measured using a commercially available kit (PG CLEIA; Fujirebio Inc., Tokyo, Japan) based on a chemiluminescence enzyme immunoassay. The pepsinogen I/II ratio was then calculated.

### 2.5 Data Analysis

Age, pathological scores, and gastrin and pepsinogen levels are expressed as means ± standard deviations (SDs). Comparisons of sex, endoscopic diseases, *H. pylori* status, and pathological parameter positivity between countries and of sex, *H. pylori* status, and pathological parameter positivity between endoscopic diseases were made using Fisher's exact test. Statistically significant differences in mean age, pathological scores, gastrin levels, and pepsinogen levels among countries or among endoscopic disease groups were assessed using one‐way ANOVA, followed by the Scheffé post hoc multiple comparison test. We decided the cut‐off values of the high‐risk group for pathological atrophy, *H. pylori* , and gastric cancer using the Youden index (maximum value after calculation [sensitivity + specificity‐1]) analyzed by receiver‐operating characteristic analysis using composite variable combined with serum pepsinogen I level and pepsinogen I/II ratio. Adjustments for multiple comparisons have not been performed. All *p*‐values were two‐sided, and *p* < 0.05 was considered statistically significant. Calculations were performed with SPSS (version 29, IBM Inc.; Armonk NY, USA).

## 3 Results

### 3.1 Patient Characteristics

A total of 2426 patients, including 608 from Vietnam, 433 from Thailand, 468 from Myanmar, 418 from Bangladesh, and 499 from Nepal, were enrolled (Table 1). The mean age was 42.1 ± 15.3 years, with 52.9% males. Although *H. pylori* infection status indicated 37.9% (95% confidence interval [CI]: 35.9%–39.8%) non‐infection, 3.9% (3.1%–4.7%) past‐infection, and 58.2% (56.3%–60.2%) current infection, the rate of normal endoscopic results or gastritis without atrophy or intestinal metaplasia was > 70%. Despite 62.1% of patients having either current or past infection, rates of pathological atrophy and intestinal metaplasia were low (Table 1). Mean chronic inflammation and neutrophil activity scores were 1.7 ± 0.9 and 1.4 ± 1.3, respectively (Table 2). Mean pathological atrophy and intestinal metaplasia scores were very low (close to zero). In patients with current *H. pylori* infection, mean atrophy and intestinal metaplasia scores were 0.0–0.1 irrespective of biopsy specimen site (Table 3).

**TABLE 1:** Characteristics of patients in five South and Southeast Asian countries.

	Total	Vietnam	Thailand	Myanmar	Bangladesh	Nepal	*p*
Patient number (*n*)	2426	608	433	468	418	499	
Age (years, mean ± SD)	42.1 ± 15.3	41.2 ± 16.3	55.2 ± 14.3	40.9 ± 12.0	36.1 ± 12.3	37.8 ± 13.5	< 0.001
Sex (*n*, male/female)	1284/1142	303/305	138/295	327/141	205/213	311/188	< 0.001
Endoscopic diseases, *n* (%)							< 0.001
Normal (Non‐gastritis)	1267 (52.2%, 95% CI: 50.2%–54.2%)	280 (46.1%, 95% CI: 42.0%–50.1%)	206 (47.6%, 95% CI: 42.8%–52.4%)	293 (62.6%, 95% CI: 58.0%–67.0%)	247 (59.1%, 95% CI: 54.2%–63.8%)	331 (66.6%, 95% CI: 62.0%–70.5%)	
Gastritis	468 (19.2%, 95% CI: 17.7%–20.9%)	113 (18.6%, 95% CI: 15.6%–21.9%)	80 (18.5%, 95% CI: 14.9%–22.5%)	102 (21.8%, 95% CI: 18.1%–25.8%)	78 (18.7%, 95% CI: 15.0%–22.7%)	95 (19.0%, 95% CI: 15.7%–22.8%)	
Atrophic gastritis	198 (8.2%, 95% CI: 7.1%–9.3%)	59 (9.7%, 95% CI: 7.5%–12.3%)	61 (14.1%, 95% CI: 11.0%–17.7%)	35 (7.5%, 95% CI: 5.3%–10.2%)	30 (7.2%, 95% CI: 4.9%–10.1%)	13 (2.6%, 95% CI: 1.4%–4.4%)	
Gastric polyp	19 (0.8%, 95% CI: 0.5%–1.2%)	0 (0.0%, 95% CI: 0.0%–0.5%)	0 (0.0%, 95% CI: 0.0%–0.7%)	6 (1.3%, 95% CI: 0.5%–2.8%)	6 (1.4%, 95% CI: 0.5%–3.1%)	7 (1.4%, 95% CI: 0.6%–2.9%)	
Peptic ulcer	437 (18.0%, 95% CI: 16.5%–19.6%)	139 (22.9%, 95% CI: 19.6%–26.4%)	81 (18.7%, 95% CI: 15.1%–22.7%)	115 (24.6%, 95% CI: 20.7%–28.7%)	51 (12.2%, 95% CI: 9.2%–15.7%)	51 (10.2%, 95% CI: 7.75%–13.2%)	
Gastric cancer	37 (1.5%, 95% CI: 1.1%–2.1%)	17 (2.8%, 95% CI: 1.6%–4.4%)	5 (1.2%, 95% CI: 0.4%–2.7%)	7 (1.5%, 95% CI: 0.6%–3.1%)	6 (1.4%, 95% CI: 0.5%–3.1%)	2 (0.4%, 95% CI: 0.0%–1.4%)	
*H. pylori* infection status, *n* (%)							< 0.001
Negative (non‐infection)	919 (37.9%, 95% CI: 35.9%–39.8%)	273 (44.9%, 95% CI: 40.9%–49.0%)	103 (23.8%, 95% CI: 19.9%–28.1%)	170 (36.3%, 95% CI: 32.0%–40.9%)	149 (35.6%, 95% CI: 31.1%–40.4%)	224 (44.9%, 95% CI: 40.5%–49.4%)	
Negative (past infection)	94 (3.9%, 95% CI: 3.1%–4.7%)	47 (7.7%, 95% CI: 5.7%–10.1%)	8 (1.8%, 95% CI: 0.8%–3.6%)	13 (2.8%, 95% CI: 1.5%–4.7%)	11 (2.6%, 95% CI: 1.3%–4.7%)	15 (3.0%, 95% CI: 1.7%–4.9%)	
Positive (current infection)	1413 (58.2%, 95% CI: 56.3%–60.2%)	288 (47.4%, 95% CI: 43.3%–51.4%)	322 (74.4%, 95% CI: 70.0%–78.4%)	285 (60.9%, 95% CI: 56.3%–65.3%)	258 (61.7%, 95% CI: 56.9%–66.4%)	260 (52.1%, 95% CI: 47.6%–56.6%)	
Pathological gastritis evaluation, *n* (%)							
Chronic inflammation	2299 (94.8%, 95% CI: 93.8%–95.6%)	595 (97.9%, 95% CI: 96.3%–98.9%)	384 (88.7%, 95% CI: 85.3%–91.5%)	443 (94.7%, 95% CI: 89.8%–94.7%)	410 (98.1%, 95% CI: 96.3%–99.2%)	467 (93.6%, 95% CI: 91.1%–95.6%)	< 0.001
Neutrophil activity	1478 (60.9%, 95% CI: 58.9%–62.92%)	327 (53.8%, 95% CI: 49.7%–57.8%)	322 (74.4%, 95% CI: 70.0%–78.4%)	281 (60.0%, 95% CI: 55.4%–64.5%)	274 (65.6%, 95% CI: 60.8%–70.1%)	274 (54.9%, 95% CI: 50.4%–59.3%)	< 0.001
Atrophy	224 (9.7%, 95% CI: 8.1%–10.5%)	91 (15.4%, 95% CI: 12.2%–18.1%)	72 (20.5%, 95% CI: 13.2%–20.5%)	27 (5.8%, 95% CI: 3.8%–8.3%)	3 (0.7%, 95% CI: 0.1%–2.1%)	31 (6.2%, 95% CI: 4.3%–8.7%)	< 0.001
Intestinal metaplasia	199 (8.2%, 95% CI: 7.1%–9.4%)	67 (11.1%, 95% CI: 8.6%–13.8%)	51 (11.8%, 95% CI: 8.9%–15.2%)	36 (7.7%, 95% CI: 5.4%–10.5%)	17 (4.1%, 95% CI: 2.4%–6.4%)	28 (5.6%, 95% CI: 3.8%–8.0%)	< 0.001
*H. pylori*	1398 (57.6%, 95% CI: 55.6%–59.6%)	288 (47.4%, 95% CI: 43.3%–51.4%)	322 (74.4%, 95% CI: 70.0%–78.4%)	274 (58.5%, 95% CI: 53.9%–63.1%)	254 (60.8%, 95% CI: 55.9%–65.5%)	260 (52.1%, 95% CI: 47.6%–56.6%)	< 0.001

Abbreviations: CI, confidence interval; *H. pylori* , *Helicobacter pylori* ; SD, standard deviation.

**TABLE 2:** Pathological evaluations by biopsy site according to the updated version of the Sydney system.

	Total	Vietnam	Thailand	Myanmar	Bangladesh	Nepal	*p*
Chronic inflammation score							
Greater curvature of antrum	1.7 ± 0.9	1.9 ± 0.9	2.0 ± 1.1	1.7 ± 0.8	1.5 ± 0.6	1.6 ± 0.8	< 0.001
Greater curvature of upper corpus	1.3 ± 0.8	1.4 ± 0.9	1.4 ± 1.0	1.2 ± 0.6	1.2 ± 0.6	1.1 ± 0.7	< 0.001
Lesser curvature of lower corpus	1.5 ± 0.9	1.6 ± 0.9	1.7 ± 1.1	1.5 ± 0.8	1.5 ± 0.7	1.4 ± 0.8	< 0.001
Neutrophil activity score							
Greater curvature of antrum	1.4 ± 1.3	1.3 ± 1.4	1.8 ± 1.3	1.3 ± 1.2	1.2 ± 1.0	1.2 ± 1.2	< 0.001
Greater curvature of upper corpus	0.9 ± 1.0	0.9 ± 1.1	1.2 ± 1.2	0.9 ± 0.9	1.0 ± 0.9	0.8 ± 0.9	< 0.001
Lesser curvature of lower corpus	1.2 ± 1.2	1.1 ± 1.2	1.5 ± 1.3	1.1 ± 1.1	1.2 ± 1.0	1.0 ± 1.1	< 0.001
Atrophy score							
Greater curvature of antrum	0.1 ± 0.4	0.2 ± 0.4	0.2 ± 0.5	0.0 ± 0.3	0.0 ± 0.1	0.0 ± 0.2	< 0.001
Greater curvature of upper corpus	0.0 ± 0.1	0.0 ± 0.1	0.0 ± 0.2	0.0 ± 0.1	0.0 ± 0.0	0.0 ± 0.0	< 0.001
Lesser curvature of lower corpus	0.1 ± 0.4	0.1 ± 0.4	0.3 ± 0.6	0.1 ± 0.4	0.0 ± 0.1	0.0 ± 0.3	< 0.001
Intestinal metaplasia score							
Greater curvature of antrum	0.1 ± 0.4	0.1 ± 0.4	0.1 ± 0.4	0.1 ± 0.4	0.0 ± 0.3	0.0 ± 0.3	< 0.001
Greater curvature of upper corpus	0.0 ± 0.1	0.0 ± 0.1	0.0 ± 0.2	0.0 ± 0.1	0.0 ± 0.0	0.0 ± 0.1	0.176
Lesser curvature of lower corpus	0.1 ± 0.4	0.1 ± 0.4	0.1 ± 0.5	0.1 ± 0.4	0.0 ± 0.3	0.0 ± 0.3	0.047
*H. pylori* score							
Greater curvature of antrum	1.3 ± 1.2	1.0 ± 1.3	1.7 ± 1.3	1.3 ± 1.2	1.1 ± 1.0	1.1 ± 1.2	< 0.001
Greater curvature of upper corpus	1.0 ± 1.1	0.9 ± 1.2	1.4 ± 1.2	1.0 ± 1.0	1.0 ± 1.0	0.8 ± 0.9	< 0.001
Lesser curvature of lower corpus	1.2 ± 1.2	1.0 ± 1.2	1.6 ± 1.2	1.2 ± 1.1	1.2 ± 1.1	1.0 ± 1.1	< 0.001

*Note:* Dara was shown as mean ± standard deviation.

Abbreviation: *H. pylori* , *Helicobacter pylori* .

**TABLE 3:** Pathological evaluations according to the updated version of Sydney system in *H. pylori* positive patients [19].

	Total	Vietnam	Thailand	Myanmar	Bangladesh	Nepal	*p*
Chronic inflammation score							
Greater curvature of antrum	2.3 ± 0.7	2.7 ± 0.5	2.5 ± 0.8	2.1 ± 0.6	1.8 ± 0.5	2.2 ± 0.6	< 0.001
Greater curvature of upper corpus	1.6 ± 0.7	1.9 ± 0.7	1.7 ± 0.9	1.5 ± 0.6	1.5 ± 0.5	1.5 ± 0.6	< 0.001
Lesser curvature of lower corpus	2.0 ± 0.8	2.3 ± 0.7	2.1 ± 0.9	1.9 ± 0.7	1.8 ± 0.6	1.9 ± 0.7	< 0.001
Neutrophil activity score							
Greater curvature of antrum	2.2 ± 0.8	2.6 ± 0.7	2.4 ± 1.0	2.1 ± 0.8	1.9 ± 0.6	2.2 ± 0.8	< 0.001
Greater curvature of upper corpus	1.5 ± 0.9	1.8 ± 0.9	1.6 ± 1.1	1.4 ± 0.8	1.5 ± 0.6	1.4 ± 0.8	< 0.001
Lesser curvature of lower corpus	1.9 ± 0.9	2.2 ± 0.9	2.0 ± 1.1	1.8 ± 0.9	1.9 ± 0.7	1.9 ± 0.9	< 0.001
Atrophy score							
Greater curvature of antrum	0.1 ± 0.4	0.3 ± 0.5	0.3 ± 0.6	0.0 ± 0.2	0.0 ± 0.0	0.0 ± 0.3	< 0.001
Greater curvature of upper corpus	0.0 ± 0.1	0.0 ± 0.1	0.0 ± 0.2	0.0 ± 0.1	0.0 ± 0.0	0.0 ± 0.1	0.006
Lesser curvature of lower corpus	0.1 ± 0.5	0.2 ± 0.5	0.4 ± 0.7	0.1 ± 0.4	0.0 ± 0.1	0.1 ± 0.3	< 0.001
Intestinal metaplasia score							
Greater curvature of antrum	0.1 ± 0.4	0.1 ± 0.5	0.1 ± 0.5	0.1 ± 0.3	0.0 ± 0.2	0.1 ± 0.3	< 0.001
Greater curvature of upper corpus	0.0 ± 0.1	0.0 ± 0.1	0.0 ± 0.1	0.0 ± 0.1	0.0 ± 0.0	0.0 ± 0.1	0.402
Lesser curvature of lower corpus	0.1 ± 0.4	0.1 ± 0.5	0.1 ± 0.5	0.1 ± 0.4	0.0 ± 0.2	0.0 ± 0.3	0.02
*H. pylori* score							
Greater curvature of antrum	2.1 ± 0.8	2.3 ± 0.9	2.3 ± 0.9	2.1 ± 0.8	1.9 ± 0.7	2.1 ± 0.7	< 0.001
Greater curvature of upper corpus	1.7 ± 0.9	2.0 ± 0.9	1.9 ± 1.0	1.6 ± 0.8	1.6 ± 0.8	1.6 ± 0.7	< 0.001
Lesser curvature of lower corpus	2.0 ± 0.8	2.2 ± 0.9	2.1 ± 1.0	1.9 ± 0.8	2.0 ± 0.7	1.9 ± 0.8	< 0.001

*Note:* Data was shown as mean ± standard deviation.

Abbreviation: *H. pylori* , *Helicobacter pylori.*

### 3.2 Differences Among South and Southeast Asian Populations

In Thailand, the mean age along with the rates of current infection (74.4%, 95% CI: 70.0%–78.4%), endoscopic atrophic gastritis (14.1%, 95% CI: 11.0%–17.7%), and pathological atrophy (20.5%, 95% CI: 13.2%–20.5%) were higher than those of other Asian countries (current infection: 47.4%–60.9%, endoscopic atrophy: 2.6%–9.7%, and pathological atrophy: 0.7%–15.4%) (Table 1). In South Asian populations in Bangladesh and Nepal, although *H. pylori* infection rates were 61.7% (95% CI: 56.9%–66.4%) and 52.1% (95% CI: 47.6%–56.6%), respectively, rates of normal endoscopic results without gastritis, erosion, or atrophy were approximately 59.1% (95% CI: 54.2%–63.8%) and 66.6% (62.0%–70.5%). The rates of atrophic gastritis and peptic ulcer were lower in these countries than those in Southeast Asian countries (Table 1).

Although mean atrophy and intestinal metaplasia scores significantly differed among different countries, they were < 0.2 in all countries, irrespective of biopsy site (Table 2). In Thailand, the mean neutrophil activity score was higher than that in other countries. In patients with current infection, mean atrophy and intestinal metaplasia scores were less than 0.3 ± 0.6 and 0.1 ± 0.5, respectively, irrespective of different countries and biopsy site (Table 3). The mean atrophy score in the Southeast Asian population was significantly higher than in the South Asian population.

### 3.3 Serological Markers of Gastric Mucosal Atrophy

Fasting serum gastrin levels ranged from 30 to 150 pg/mL, but mean gastrin levels at the fasting time varied widely among Asian countries, with Myanmar having the highest value of 138.2 ± 136.0 pg/mL and Thailand having the lowest (67.1 ± 31.8 pg/mL) (Table 4). Mean serum pepsinogen I/II levels and ratios significantly differed between countries (Table 4).

**TABLE 4:** Serum markers of atrophy.

	Total	Vietnam	Thailand	Myanmar	Bangladesh	Nepal	*p*
Gastrin (mean ± SD)	120.2 ± 119.4	82.1 ± 71.2	67.1 ± 31.8	138.2 ± 136.0	163.0 ± 142.2	112.7 ± 111.7	< 0.001
Pepsinogen I (mean ± SD)	71.8 ± 64.5	67.8 ± 71.8	69.3 ± 37.9	76.2 ± 51.0	5.5 ± 62.9	70.7 ± 84.1	< 0.001
Pepsinogen II (mean ± SD)	17.5 ± 15.5	15.5 ± 16.2	15.8 ± 9.5	19.7 ± 17.0	18.1 ± 145.5	18.6 ± 17.0	< 0.001
Pepsinogen I/II ratio (mean ± SD)	4.6 ± 1.7	4.9 ± 1.7	5.0 ± 1.9	4.3 ± 1.6	4.4 ± 1.6	4.2 ± 1.6	< 0.001

Abbreviation: SD, standard deviation.

**FIGURE 1:**
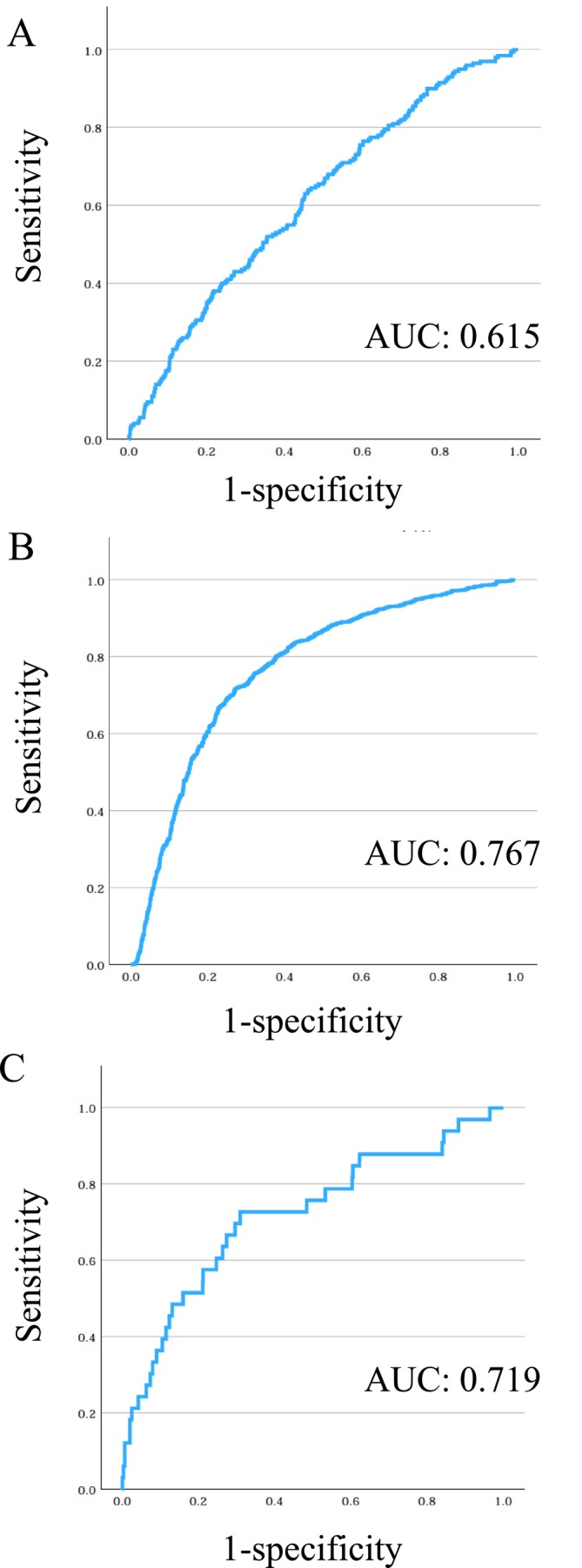
Receiver‐operating characteristic analysis using serum pepsinogen I level and pepsinogen I/II ratio for selection of patients with pathological atrophy (A), infected with *H. pylori* (B), and with gastric cancer (C). Abbreviation: AUC, are under the curve.

Histopathological atrophy, *H. pylori* infection, and gastric cancer development using serum marker of pepsinogen (serum pepsinogen I level and pepsinogen I/II ratio) were designated “High pathological atrophy” (Youden index: > 0.179), “High *H. pylori* infection” (Youden index: > 0.445), and “High gastric cancer development” (Youden index: > 0.417), respectively (Figure 1). The areas under the curves (AUCs) of the higher risk groups for *H. pylori* infection and gastric cancer development were 0.767 and 0.719, respectively, indicating there is a possibility of diagnosing *H. pylori* infection and gastric cancer development using serum pepsinogen I level and pepsinogen I/II ratio in South and Southeast Asian populations (Figure 1).

### 3.4 Associations Between Gastric Diseases and *H. pylori* in South and Southeast Asian Populations

The mean age in patients with gastric cancer was 54.0 ± 16.5, which was significantly higher than those for other diseases (Table 5). Among patients diagnosed endoscopically as normal, 2.1% (95% CI: 1.3%–3.0%) had past infection and 53.4% (95% CI: 50.6%–56.1%) had current infection, while 30.3% (95% CI: 24.0%–37.2%) of patients with atrophic gastritis were in the non‐infection group. Non‐infection rates in gastric polyp, peptic ulcer, and gastric cancer were 36.8% (95% CI: 16.3%–61.6%), 17.2% (13.7%–21.0%) and 35.1% (20.2%–52.5%), respectively. In histopathological evaluation, positive rates of atrophy and intestinal metaplasia in patients with gastric cancer were 22.6% (95% CI: 8.0%–35.2%) and 45.9% (95% CI: 29.5%–63.1%), respectively, significantly higher than in other diseases. Weak correlation was observed with endoscopic atrophy [atrophic gastritis vs. non‐atrophic gastritis (normal and gastritis)] and pathological atrophy (> 1 according to the Sydney system in South and Southeast Asian populations) (*γ*: 0.213, *p* < 0.001).

**TABLE 5:** Characteristics of gastric diseases in South and Southeast Asian populations.

	Total	Normal	Gastritis	Atrophic gastritis	Gastric polyp	Peptic ulcer	Gastric cancer	*p*
Patient number (*n*)	2426	1267	468	198	19	437	37	
Age (years, mean ± SD)	42.1 ± 15.3	39.5 ± 14.9	42.9 ± 14.6	48.3 ± 15.8	40.8 ± 13.5	44.9 ± 15.2	54.0 ± 16.5	< 0.001
Sex (male/female)	1284/1142	644/623	248/220	76/122	6/13	289/148	21/16	< 0.001
*H. pylori* infection status, *n* (%)								< 0.001
Negative (non‐infection)	919 (37.9%, 95% CI: 35.9%–59.8%)	565 (44.6%, 95% CI: 41.8%–47.4%)	199 (42.4%, 95% CI: 38.0%–47.1%)	60 (30.3%, 95% CI: 24.0%–37.2%)	7 (36.8%, 95% CI: 16.3%–61.6%)	75 (17.2%, 95% CI: 13.7%–21.0%)	13 (35.1%, 95% CI: 20.2%–52.5%)	
Negative (past infection)	94 (3.9%, 95% CI: 3.1%–4.7%)	26 (2.1%, 95% CI: 1.3%–3.0%)	18 (3.9%, 95% CI: 2.3%–6.0%)	18 (9.1%, 95% CI: 5.5%–14.0%)	3 (15.8%, 95% CI: 3.4%–39.6%)	23 (5.3%, 95% CI: 3.4%–7.8%)	6 (16.2%, 95% CI: 6.2%–32.0%)	
Positive (current infection)	1413 (58.2%, 95% CI: 56.3%–60.2%)	676 (53.4%, 95% CI: 50.6%–56.1%)	251 (53.7%, 95% CI: 49.0%–58.2%)	120 (60.6%, 95% CI: 53.4%–67.1%)	9 (47.4%, 95% CI: 24.5%–71.1%)	339 (77.6%, 95% CI: 73.4%–81.4%)	18 (48.6%, 95% CI: 31.9%–65.6%)	
Pathological evaluation, *n* (%)								
Chronic inflammation	2299 (94.8%, 95% CI: 93.8%–95.6%)	1186 (93.6%, 95% CI: 92.1%–94.9%)	441 (94.2%, 95% CI: 91.7%–96.1%)	191 (96.5%, 95% CI: 92.9%–98.6%)	15 (78.9%, 95% CI: 54.4%–93.0%)	432 (98.9%, 95% CI: 87.4%–99.6%)	34 (91.9%, 95% CI: 78.1%–98.3%)	< 0.001
Neutrophil activity	1478 (60.9%, 95% CI: 58.9%–62.9%)	700 (55.2%, 95% CI: 52.5%–58.0%)	265 (56.7%, 95% CI: 52.0%–61.2%)	126 (63.6%, 95% CI: 56.5%–70.3%)	11 (57.9%, 95% CI: 33.5%–79.7%)	353 (80.8%, 95% CI: 76.8%–84.4%)	23 (62.2%, 95% CI: 44.8%–77.5%)	< 0.001
Atrophy	224 (9.7%, 95% CI: 8.1%–10.5%)	71 (5.9%, 95% CI: 4.4%–7.0%)	43 (9.5%, 95% CI: 6.7%–12.2%)	50 (27.0%, 95% CI: 19.4%–31.9%)	2 (10.5%, 95% CI: 1.3%–33.1%)	51 (12.0%, 95% CI: 8.8%–15.1%)	7 (22.6%, 95% CI: 8.0%–35.2%)	< 0.001
Intestinal metaplasia	199 (8.2%, 95% CI: 7.1%–9.4%)	59 (4.7%, 95% CI: 3.6%–6.0%)	32 (6.9%, 95% CI: 4.7%–9.5%)	37 (18.8%, 95% CI: 13.5%–24.8%)	2 (10.5%, 95% CI: 1.3%–33.1%)	52 (11.9%, 95% CI: 9.0%–15.3%)	17 (45.9%, 95% CI: 29.5%–63.1%)	< 0.001
*H. pylori*	1398 (57.6%, 95% CI: 54.6%–59.6%)	664 (52.4%, 95% CI: 49.6%–55.1%)	250 (53.5%, 95% CI: 48.8%–58.0%)	119 (60.1%, 95% CI: 52.9%–67.0%)	9 (47.4%, 95% CI: 24.0%–37.2%)	338 (77.3%, 95% CI: 73.1%–81.2%)	18 (48.6%, 95% CI: 31.9%–65.6%)	< 0.001
Serum marker (mean ± SD)								
Gastrin	120.2 ± 119.4	123.6 ± 118.5	109.1 ± 93.2	140.4 ± 183.3	125.72 ± 75.7	111.4 ± 106.1	160.4 ± 203.0	0.142
Pepsinogen I	71.8 ± 64.5	69.5 ± 67.0	69.2 ± 71.8	68.3 ± 50.5	45.2 ± 19.5	82.7 ± 53.4	85.6 ± 67.8	< 0.001
Pepsinogen II	17.5 ± 15.5	16.3 ± 14.15	16.65 ± 15.4	16.9 ± 12.4	11.6 ± 6.6	20.7 ± 14.3	35.2 ± 47.1	< 0.001
Pepsinogen I/II ratio	4.6 ± 1.7	4.7 ± 1.7	4.5 ± 1.7	4.5 ± 1.7	4.5 ± 1.7	4.4 ± 1.5	3.4 ± 1.6	< 0.001

Abbreviations: CI, confidence interval; *H. pylori* , *Helicobacter pylori* ; SD, standard deviation.

### 3.5 Characteristics of Patients Among Different *H. pylori* Infection Status

The ratio of endoscopic diseases significantly differed among different *H. pylori* infection statuses, with more patients of atrophic gastritis, peptic ulcer, and gastric cancer observed in the current infection group and past infection group than in the non‐infection group (Table 6). The past infection group was older than the other groups and had a higher positive rate of pathological atrophy and intestinal metaplasia.

**TABLE 6:** Characteristics of patients among different *H. pylori* infection statuses.

	Current infection	Non infection	Past infection	*p*
Patient number (*n*)	1413	919	94	
Age (years, mean ± SD)	42.5 ± 15.3	40.7 ± 15.1	49.4 ± 15.3	< 0.001
Sex (*n*, male/female)	776/637	460/459	48/46	0.021
Endoscopic diseases, *n* (%)				< 0.001
Normal (Non‐gastritis)	676 (47.8%, 95% CI: 45.2%–50.5%)	565 (61.5%, 95% CI: 58.2%–64.6%)	26 (27.7%, 95% CI: 18.9%–37.8%)	
Gastritis	251 (17.8%, 95% CI: 15.8%–19.9%)	199 (21.6%, 95% CI: 19.0%–24.5%)	19 (19.1%, 95% CI: 12.6%–29.8%)	
Atrophic gastritis	120 (8.5%, 95% CI: 7.1%–10.1%)	60 (6.5%, 95% CI: 5.0%–8.3%)	18 (19.1%, 95% CI: 11.8%–28.6%)	
Gastric polyp	9 (0.6%, 95% CI: 0.3%–1.2%)	7 (0.8%, 95% CI: 0.3%–1.6%)	3 (3.2%, 95% CI: 0.7%–9.0%)	
Peptic ulcer	339 (24.0%, 95% CI: 21.8%–26.3%)	75 (8.2%, 95% CI: 6.4%–10.1%)	23 (24.5%, 95% CI: 16.2%–34.4%)	
Gastric cancer	18 (1.3%, 95% CI: 0.8%–2.0%)	13 (1.4%, 95% CI: 0.8%–2.7%)	6 (6.4%, 95% CI: 2.4%–13.4%)	
Pathological gastritis evaluation, *n* (%)				
Chronic inflammation	1406 (99.5%, 95% CI: 99.0%–99.8%)	802 (87.3%, 95% CI: 84.9%–89.4%)	91 (96.8%, 95% CI: 91.0%–99.3%)	< 0.001
Neutrophil activity	1381 (97.7%, 95% CI: 96.8%–98.4%)	78 (8.5%, 95% CI: 6.8%–10.5%)	19 (20.2%, 95% C 12.6%–29.8%)	< 0.001
Atrophy	172 (12.9%, 95% CI: 10.5%–14.0%)	0 (0%, 95% CI: 0%–0.3%)	52 (56.5%, 95% CI: 44.7%–65.6%)	< 0.001
Intestinal metaplasia	133 (9.4%, 95% CI: 7.9%–11.1%)	0 (0%, 95% CI: 0%–0.3%)	66 (70.2%, 95% CI: 59.9%–79.2%)	< 0.001
Gastrin	113.2 ± 96.8	128.6 ± 143.2	127.9 ± 153.9	< 0.001
Pepsinogen I (mean ± SD)	73.8 ± 48.2	67.6 ± 72.8	82.8 ± 141.5	< 0.001
Pepsinogen II (mean ± SD)	20.0 ± 13.0	13.5 ± 16.0	18.8 ± 30.0	< 0.001
Pepsinogen I/II ratio (mean ± SD)	4.0 ± 1.4	5.4 ± 1.7	4.9 ± 1.9	< 0.001

Compared with the *H. pylori* non‐infection group, the risks of peptic ulcer and gastric cancer were 3.55 (95% CI: 2.72–4.63) and 0.90 (0.44–1.84) in the current infection group and 3.65 (2.15–6.17) and 4.75 (1.76–12.8) in the past‐infection group (Table 7).

**TABLE 7:** Factor for determining *H. pylori* non‐infection status.

	Current infection			Past infection		
	OR	95% CI	*p*	OR	95% CI	*p*
Endoscopic diseases						
Gastritis	0.78	0.64–0.96	0.020	0.86	0.50–1.47	0.573
Atrophic gastritis	1.33	0.96–1.83	0.083	3.39	1.905–6.035	< 0.001
Gastric polyp	0.84	0.31–2.25	0.722	4.30	1.09–16.90	0.037
Peptic ulcer	3.55	2.72–4.63	< 0.001	3.65	2.15–6.17	< 0.001
Gastric cancer	0.90	0.44–1.84	0.722	4.75	1.76–12.8	0.002
Pathological gastritis evaluation						
Chronic inflammation	29.30	13.60–63.13	< 0.001	4.43	1.38–14.21	0.012
Neutrophil activity	465.31	305.64–708.39	< 0.001	2.73	1.57–4.76	< 0.001
Atrophy	—	—	—	—	—	—
Intestinal metaplasia	—	—	—	—	—	—

*Note:* OR versus *H. pylori* non‐infection.

## 4 Discussion

We investigated the severity of endoscopic and pathological gastritis using the same endoscopic and histopathological criteria in South and Southeast Asians. Although the mean pathological atrophy and intestinal score significantly differed between countries, there was a low incidence and low severity of atrophy and intestinal metaplasia, even in those with current and past infections. This is likely to impact the incidence of gastric cancer and requires careful assessment of gastric cancer risk, including ethnicity, living conditions, and the virulence of *H. pylori* strains.

### 4.1 Differences Among South and Southeast Asian Countries

The severity of gastritis is influenced by diet, genetic factors, *H. pylori* virulence, lifestyle, and chemical exposure. Patients infected with highly virulent *H. pylori* genotypes are at risk of severe gastric mucosal damage and atrophy, intestinal metaplasia, peptic ulcer, and gastric cancer. Although many factors are related to high virulence, *cagA* and *vacA* are major virulence factors of *H. pylori* . The *vacA* s1, m1, and i1 genotypes and *cagA*‐positive strains cause elevated inflammatory cell infiltration compared with that induced by *vacA* s2, m2, and i2 genotypes and *cagA*‐negative strains, increasing the risk of peptic ulcer and gastric cancer [20, 21]. We previously performed a meta‐analysis of 14 studies with 1355 *H. pylori* strains detected in Southeast Asian countries, finding the rates of *vacA* genotypes, *cagA* status, and C‐terminal Glu‐Pro‐Ile‐Tyr‐Ala motif type differed significantly between Southeast Asian countries, ethnic groups in the same country, and gastric diseases [10]. Although *H. pylori* virulence factors were not assessed in the present study, in South and Southeast Asian countries, fewer patients were infected with *H. pylori* strains showing high virulence to gastric mucosa compared with East Asian populations and other populations with high gastric cancer prevalence [22]. It has been reported that even in the same South and Southeast Asian countries, incidence rates of *H. pylori* ‐related diseases vary in correlation with the rate of highly virulent *H. pylori* genotypes and that disease incidence rates were lower than those in East Asian populations and other populations with high gastric cancer prevalence.

GLOBOCAN 2022 data showed that gastric cancer is the 5th most common cancer (new cases: 968350; % of all sites: 4.9%) and the 5th most deadly [23]. The incidence of gastric cancer varies across regions, with South and Southeast Asia having lower incidences (age‐standardized incidence rate per 100 000: 7.3 and 6.7, respectively) compared with East Asia (23.0) [23]. Although gastric cancer associated with continuous long‐term *H. pylori* infection is caused by a multifactorial process including atrophy and intestinal metaplasia [2, 3, 4], this study showed that mean pathological atrophy and intestinal metaplasia scores were 0.0–0.1 in patients with current infection, irrespective of biopsy site. Although this may be influenced by differences in *H. pylori* strain virulence factors, as mentioned above, South Asia and Southeast Asian populations generally had a lower severity of pathological atrophy and intestinal metaplasia, which may be responsible for the lower incidence of gastric cancer.

### 4.2 Pepsinogen Test and Serum Gastrin Level as an Atrophy Marker in South and Southeast Asian Countries

Serum pepsinogen is a biomarker for predicting the status of gastric mucosa, and pepsinogen I level and I/II ratio are established evaluation markers for atrophy severity [24, 25, 26]. Because *H. pylori* and atrophy are risk factors of gastric cancer, the ABC method classifies the risk for gastric cancer into four groups based on combinations of pepsinogen and *H. pylori* positivity [24]; this method is used at health check‐ups to identify patients at higher risk of gastric cancer in Japan. Patients in ABC method group A are advised to have endoscopic check‐ups every 5 years, those in group B every 3 years, those in group C every 2 years, and those in group D annually, to detect gastric cancer at an early stage [24].

Pepsinogen‐positive patients are at nearly 10‐fold increased risk for developing gastric cancer compared with pepsinogen‐negative patients. In Croatia, when pepsinogen‐positive cutoff values were pepsinogen I ≤ 70 μg/L and pepsinogen I/II ratio < 3, the accuracy, sensitivity, and specificity were determined to be 87.2%, 78.1%, and 90.1%, respectively, for gastric cancer diagnosis [27]. In a meta‐analysis including 31 studies with 1520 patients with gastric cancer, especially in Asian and European studies, pepsinogen positivity demonstrated a pooled sensitivity for gastric cancer of 0.69 (95% confidence interval [CI]: 0.60–0.76) and a pooled specificity of 0.73 (95% CI: 0.62–0.82) [28]. Pepsinogen I levels ≤ 70 μg/L and a pepsinogen I/II ratio < 3 may be considered globally applicable.

In this study, mean serum pepsinogen I level, pepsinogen II level, and pepsinogen I/II ratio significantly differed among South and Southeast Asian countries; the mean pepsinogen I level and pepsinogen I/II ratio across all countries were 71.8 ± 64.5 and 4.6 ± 1.7, respectively (Table 4). Although patients with gastric cancer generally have severe atrophy, mean pepsinogen I level and pepsinogen I/II ratio in patients with gastric cancer were 85.6 ± 67.8 and 3.4 ± 1.6, respectively, higher than those in previous reports. This may be a specific characteristic in these districts. The association between pepsinogen measurement and gastric cancer is useful in previous studies in East Asian and Western populations but has not been established in South and Southeast Asian populations. In this study, when we designated a higher‐risk group for gastric cancer using the Youden index, pepsinogen I level and pepsinogen I/II ratio, the AUC was 0.719. Although pepsinogen I level and pepsinogen I/II ratio may be useful for identifying patients with gastric cancer in South and Southeast Asian populations, the cut‐off values may need to be set independently.

Serum gastrin levels are generally elevated in patients with gastrin‐producing tumors (neuroendocrine tumors), severe gastric atrophy due to autoimmune gastritis (AIG) or *H. pylori* ‐associated gastritis, and in those receiving potent acid suppressive therapy such as proton pump inhibitors [29, 30, 31]. When these confounding factors are excluded, elevated gastrin levels can serve as an indirect marker of extensive mucosal atrophy, which is itself a significant risk factor for gastric cancer development. In our study, patients with gastric cancer exhibited higher mean serum gastrin levels compared with those with other gastric conditions. This may reflect the presence of background atrophy, even when endoscopic and histological findings appear mild. Therefore, serum gastrin may serve as a potential biomarker for identifying high‐risk individuals in South and Southeast Asian populations. Further studies are warranted to validate its clinical utility in these settings.

### 4.3 Endoscopic Evaluation for Gastritis Severity in South and South‐East Asian Countries

The US and Europe have developed pathological reporting systems to identify patients at high risk of gastric cancer based on pathological gastritis severity [19, 32, 33]. Recently, the Management of epithelial precancerous conditions and lesions in the stomach II guideline stated that the status and severity of both atrophy and intestinal metaplasia should be evaluated by histopathological evaluation using the operative link on gastritis assessment and the operative link on gastritis/intestinal‐metaplasia assessment system [34]. Although endoscopic reporting systems have also been proposed for the evaluation of patients at high risk for gastric cancer based on the severity of endoscopic atrophy and intestinal metaplasia [18, 35, 36], it is important to assess whether the endoscopic evaluation is consistent with histopathologic evaluation [37, 38, 39, 40]. Endoscopic atrophy was associated with high scores for pathological atrophy using the updated Sydney system, and endoscopic intestinal metaplasia was associated with high scores of pathological intestinal metaplasia [38]. Sumi et al. PEVuZE5vdGU [40] reported that the sensitivity and specificity of endoscopic findings based on the Kyoto classification of gastritis were 98.7% and 98.4% for histological gastritis, respectively. However, we failed to show a significant correlation with endoscopic and pathological atrophy, and only a weak correlation was observed among South and Southeast Asians (*γ*: 0.213, *p* < 0.001).

Although this difference may be caused by different populations, it may also be due to the mild pathological severity of background gastric inflammation and endoscopic diffuse redness, even in patients with current infection in South and Southeast Asia. In addition, because this study used endoscopic data collected between 1996 and 2012, the image quality and diagnostic sensitivity may not reflect those of current endoscopic practice. Moreover, although recent advances in image‐enhanced endoscopy (IEE), including narrow band imaging and linked color imaging, have significantly improved the accuracy of evaluating atrophic changes and detecting gastric lesions such as early gastric cancer, such technologies were not available during the study period. Therefore, the absence of IEE may have contributed to the weak correlation between endoscopic and histopathological findings [41, 42].

In the future, it would be necessary to establish an endoscopic scoring system that combines both white light imaging and IEE to improve the assessment of gastritis severity and gastric cancer risk, especially in populations where endoscopic atrophy is subtle. While we believe that the pathological evaluation was reliable due to the use of a single experienced pathologist, subtle variability in endoscopic recognition may also have played a role in the observed discrepancy.

## 5 Limitation

This study has several limitations. First, this study used endoscopic data collected between 1996 and 2012. Since then, endoscopic technology has significantly advanced in terms of both scopes and imaging systems, enabling clearer and more detailed visualization. Therefore, the quality of the endoscopic images and the detection rate of subtle mucosal findings in this study may not fully reflect the capabilities of current endoscopic practice. Second, although it is better to provide a comparison with established thresholds at AUC values for pepsinogen markers in predicting atrophy, *H. pylori* infection, and gastric cancer from other geographic regions, we have no pepsinogen data from other regions to compare. Third, *H. pylori* infection rates and disease rates vary depending on the age and gender of each group (countries) rather than the number of patients enrolled in this study, so it may be important to conduct a study that matches these factors in order to eliminate bias. Fourth, although there have been reports of a correlation among *H. pylori* infection, gastric diseases and polyps [43], because we did not perform a pathological evaluation for gastric polyps, we cannot evaluate the correct role of gastric polyps in different *H. pylori* infection statuses.

## 6 Summary

Despite being in the same region and with a high infection rate of *H. pylori* , gastric conditions and susceptibility to gastric disease differed among South and Southeast Asian populations. In this large epidemiological study, because the frequency of atrophy and severity were low and mild in South and Southeast Asians, it may be difficult to stratify the risk of gastric cancer and diagnose *H. pylori* by endoscopy alone. It may be necessary to develop endoscopic and histopathological scoring systems that can be effectively used in areas where the frequency and severity of atrophy are low.

## Ethics Statement

The study protocol conformed to the ethical guidelines of the Declaration of Helsinki, and the ethics committees approved the conduct of this study. Ethical approval was given by the Ethics Committee of the Oita University Faculty of Medicine (P‐12‐10, and #1660).

## Consent

Because this study was conducted under a retrospective design, and written informed consent was not obtained from each enrolled patient, a document describing an opt‐out policy through which potential patients and/or relatives could refuse inclusion was uploaded on the Oita University website.

## Conflicts of Interest

The authors declare no conflicts of interest.

## Data Availability

The data that support the findings of this study are available on request from the corresponding author. The data are not publicly available due to privacy or ethical restrictions.
